# Optimizing the Management of Cancer Patients Treated With Systemic Therapies During the COVID-19 Pandemic: The New Role of PCR and CT Scan

**DOI:** 10.3389/fonc.2021.560585

**Published:** 2021-05-28

**Authors:** Alessandro A. Viansone, Samy Ammari, Laurent Dercle, Monica Arnedos

**Affiliations:** ^1^ Breast Unit, Department of Medicine, Gustave Roussy Cancer Campus, Villejuif, France; ^2^ Breast Unit–Oncology Unit, Department of Medicine, University Hospital of Parma, Parma, Italy; ^3^ Radiology Department, Gustave Roussy Cancer Campus, Villejuif, France; ^4^ Radiology Department, Columbia University Medical Center New York Presbyterian Hospital, New York, NY, United States

**Keywords:** COVID-19, PCR, CT-scan, oncology, cancer

## Abstract

In late 2019 and early 2020, the world witnessed the outbreak of the SARS-CoV-2 (also referred as COVID-19) in Wuhan, China. Its rapid expansion worldwide and its contagiousness rate have forced the activation of several measures to contain the pandemic, mostly through confinement and identification of infected patients and potential contacts by testing.

## Introduction

It is well established that patients with cancer are more susceptible to infections since they tend to be older, have multiple comorbidities, and because of the immunosuppressive state caused by anticancer treatments (1), so they could be potentially at particular risk from COVID-19 infection (2).

In a Chinese analysis (3) Professor He and colleagues found that patients with cancer presented an important risk of secondary events due to COVID-19 if we compare it with a population without cancer: 39% in the oncologic population *versus* 8%, hazard ratio of 3.56. Zhang and colleagues in a retrospective analysis identified 28 patients positive for COVID and with a cancer among 1,276 patients admitted in hospital. This prevalence (2.2%) is 1.7 (95% confidence interval, 1.2, 2.4) times higher than the Chinese population of the same age (4). The probability of dying for COVID with a cancer was of 28.6% (5). Zhang found that the administration of anticancer therapies was an independent predictor of death. They also described the high proportion of patients who acquired the infection in hospital, when they came for cancer treatment.

Although these data included a very small and heterogeneous sample of patients with cancer, reports from Italy confirm the potential higher risk of COVID-19 infection in patients with cancer, indicating that the 20% of patients dead for COVID-19 infection also had a concomitant diagnosis of cancer (6). More recently, clinical data on COVID-19 cases from two hospitals in New York City (7) observed that 23 out of the 393 (5.9%) reported cases were patients with cancer, and 10 of them required invasive mechanical ventilation (representing 7.7% of the total of patients requiring invasive mechanical ventilation). In another institution from New York City, 28% of COVID-19 + cancer patients died from COVID-19 with a case fatality rate of 37% for hematologic malignancies and 25% for solid malignancies. This study reported that in this population, older age, multiple comorbidities, need for ICU support, and elevated levels of D-dimers, LDH, and lactate predicted poorer outcome (7).

Therefore, healthcare professionals were rapidly faced with the challenge of profoundly re-organizing healthcare systems at unprecedented pace during the COVID-19 crisis to balance the competing risks of death from cancer *vs.* death or serious complications from infection.

The Chinese series suggested that postponing adjuvant chemotherapy or elective surgery for less aggressive cancers should be considered. Furthermore, more intensive care should be done for the patients with cancer who are infected (3). These measures have been later taken by healthcare organizations like ASCO and ESMO who have deepened these recommendations extending it to postponing clinics or balancing the cost/benefit ratio according to outcome, with prioritization of adjuvant therapies (8). Similarly, the benefits and risks of palliative therapies and the options of temporary stopping the therapy and switch to oral drugs, if available, during the pandemic needed to be considered. Recommendations for management of cancer patients in clinical trials have also been made available by different regulatory agencies (9). However, as this outbreak prolongs in time and with the unknown risk-benefit balance between undertreating patients with cancer (with resultant increase in cancer-related morbidity and mortality) and preventing the expected higher morbidity and mortality from COVID-19, initiation of systemic cancer therapy seems unavoidable.

The scarce of systematic information on prevalence and incidence in overall population toughen the possibility of real comparisons with patients with cancer although contagiousness rates seem rather high. For example, estimates on infection rates vary from 0.76% for residents of Iceland to 36% for residents of Boston. This likely overstates the overall population mean, which some observers have suggested is around 40% (10).

Systematic reports about the prevalence of cancer in patients with COVID-19 and the real incidence of COVID-19 infection among cancer patients are starting to be made public. A review and meta-analysis reported by Desai et al. (11) found 11 studies of patients with COVID-19 with the data of cancer prevalence: 2% (95% CI 2.0 to 3.0%: I^2^ = 83.2%) in patients treated for COVID-19. A similar meta-analysis by Emami et al. (12) reported a prevalence of malignancy of 0.92% (95% CI, 0.56–1.34%). In a recent dedicated session to cancer patients and COVID-19 infection during the last AACR annual symposium, several cancer institutions and hospitals across the world presented updates on their management and outcome of cancer patients with COVID-19 infection. These series reported variability within different countries in terms of incidence and prevalence. For example, in our own series, of Gustave Roussy Hospital, including more than 1,300 tested cancer patients, COVID-19 positivity was observed in around 12%, whereas retrospective series including 1,524 cancer patients from Wuhan reported a COVID-19 positivity rate of only 0.79% (13).

The standard method to diagnose infection by COVID-19 is through identification by RT-PCR SARS-CoV-2 testing. Since SARS-CoV-2 is usually transmitted by the upper respiratory tract, mostly swab samples are taken from the nasopharynx. It has also been accepted to perform the swabs directly to the oropharynx, although some studies suggest that the nasopharynx would be somewhat more sensitive than the oropharynx (14). Positivity from nasopharynx swabs is variable and ranges from 53.6 to 73.3% depending on the series (15). There are several factors that might affect the performance (sensitivity and specificity) of the test like the quality of the sample, the sampling technique, transportation process, or limited gene detection. In fact, it has been shown that high viral loads soon after symptom onset, which then gradually decreased towards the detection limit at about day 21, with no obvious difference in viral loads across sex, age groups, and disease severity (16). In fact, similar viral loads have been documented in the upper respiratory tract of both symptomatic and asymptomatic cases (17) and in the pre-symptomatic phase (18).

Several strategies are available in order to increase sensitivity and specificity of this testing. In a report of 67 patients with confirmed COVID-19 infection, the duration of positive test in nasopharyngeal swabs has a median of 12 days (range, 3–38), in sputum of 19 days (range, 5–37), and in stools of 18 days (range, 7–26). SARS-CoV-2 RNA was detectable for a duration of 30 days (19). After a negative test of nasopharyngeal swabs among 46 patients, 28 (60.9%) and 14 (30.4%) patients were still positive in sputum and stools.

Another approach consists in the realization of chest CT scans in patients suspected or tested for COVID-19 in addition to RT-PCR for nasal or oropharynx swabs. Consistently between patients’ series, the main symptoms associated to SARS-CoV-2 infection in addition to fever is the presence of pulmonary symptoms ranging from dry cough to pneumonia up to acute distress respiratory syndrome leading to death (3). Therefore initially it was suggested that lung cancer patients or patients who had suffered previous lung surgery would be at higher risk of lung complications from SARS-CoV-2 so more intensive follow-up and chest CT scrutiny should be required for these patients. Initial data from Wuhan series (20) indeed reported a higher risk of COVID-19 infection among their cancer patients (7 out of 28; 25%). A recent international series compilation of 200 patients with thoracic tumors affected with COVID-19 (21) reported an extremely high death rate of 34.6%, mostly due to acute respiratory distress syndrome and multi-organ failure. These findings have not been replicated by other series like Wuhan reports (22) where mortality was not affected by type of cancer (any cancer *vs.* lung cancer; HR = 0.727; p = 0.589). What it has been noteworthy is that chest CT demonstrates typical radiographic features in almost all COVID-19 patients irrespective of presence of cancer or type of cancer. These include ground-glass opacities, multifocal patchy consolidation, and/or interstitial changes with a peripheral distribution (23). Those typical pulmonary abnormalities were also observed in patients with negative RT-PCR results but clinical symptoms in small-scale studies (24).

A report of the correlation of chest CT and RT-PCR testing in 1,014 cases from China found a RT-PCR positivity in 59% (n = 601) of the patients and a chest CT positivity of 88% (n = 888). The negative RT-PCR results is correlated to a 75% (n = 308) with positive chest CT findings. The combination of RT-PCR and chest CT gave respectively a sensitivity, specificity, and accuracy of 97% (n = 580), 25% (n = 105), and 68% (n = 685), a positive predictive value of 65% (n = 580), and a negative predictive value of 83% (n = 105) (25). Another work analyzed 1,099 hospitalized patients with a positive test for COVID-19: 86% (n = 840) of patients had CT imaging with finding of ground-glass opacity, local patchy shadowing, or interstitial pneumopathies; 17.9% (n = 157) of patients has no radiographic abnormality (26).

Despite the standardized use of CT scans in addition to (or instead of) RT-PCR for the diagnoses of a suspected COVID-19 infection might still be controversial (27). First, the findings on chest imaging in COVID-19 are indeed not specific, and overlap with other infections, including influenza, H1N1, SARS, and MERS. Second, there are issues related to cleaning imaging equipment to control the spread of infection in health care facilities where CT scans are frequently used. For instance, portable radiography units are less expensive, can be cleaned easier, and could be an alternative. Chest CT scan might provide prognostic information as, some published data reported that the presence of a patchy consolidation by lung CT scan at patient admission was associated to possibility of a severe event in a multivariate analysis in COVID-19+ patients (HR = 5.438; CI 1.498–19.748; p = 0.010). In addition, it might prove useful for the management of patients with COVID-19 infection, especially in highly symptomatic cases. Indeed, as additional knowledge of this infection becomes available, several reports have shown that COVID-19 infection might be associated to an inflammatory syndrome evidenced by high levels of inflammatory markers and increased risk of thromboembolism associated to this infection (28). This might be important in patients with cancer with already increased phenomena of coagulopathy and thrombosis. Hence, the interest of associating chest imaging in cancer patients to identify underlying pulmonary embolism, which might contribute to worsen respiratory symptoms and require specific additional treatment (29). Beyond the role of CT scan without intravenous contrast agent injection for the diagnostic workup, prognostic evaluation, and follow-up of COVID-19 infection, selected patients may benefit from contrast enhanced CT pulmonary angiography to diagnose potentially life-threatening pulmonary embolism and start appropriate therapies. A study reported a high frequency of either pulmonary embolism in critically ill ICU patients with COVID-19 complications [7.1, 20.6 (30), and 49% (31)].

Rogado et al. detected 45/1,069 COVID-19 diagnoses in cancer patients *vs* 42,450/6,662,000 in total population (p < 0.00001) in a Spanish hospital. Mortality rate: 19/45 cancer patients *vs* 5,586/42,450 (p = 0.0001). Mortality was associated with older median age, adjusted by staging, and histology (74 *vs* 63.5 years old, OR 1.06, p = 0.03). Patients who combined hydroxychloroquine and azithromycin presented 3/18 deaths, regardless of age, staging, histology, cancer treatment, and comorbidities (OR 0.02, p = 0.03) (32).

In a Spanish series Mestre-Gomez et al. retrospectively reviewed 452 electronic medical records of patients admitted to Internal Medicine Department of a secondary hospital in Madrid during COVID-19 pandemic outbreak. Ninety-one patients had a Computed Tomography pulmonary angiography (CTPA). The cumulative incidence of PE was assessed with a clinical, analytical, and radiological characterization compared in patients with and without PE. The incidence of PE was 6.4%. They evaluated the D-dimer peak and they found a significant elevation in PE *vs* non-PE patients (14,480 *vs* 7,230 mcg/dl, p = 0.03). In multivariate analysis that plasma D-dimer peak was confirmed as an independent predictor of PE with a best cut off point of >5,000 µg/dl (33).

In a hematologic series at the University Hospital Freiburg, Shoumariyeh et al. analyzed a retrospective cohort of 39 patients with hematological and solid cancers hospitalized for COVID-19. With univariate and multivariate Cox analysis they found that the presence of a malignancy was not significantly associated with survival or severe events. Instead the high IL-6 levels at COVID-19 diagnosis (HR = 6.95, P = .0121) and age ≥65 years (HR = 6.22, P = .0156) were related to mortality. Another find of Shoumariyeh et al. was about patients with a hematological malignancy that showed a longer duration of clinical improvement and longer hospitalization compared to patients with a solid cancer (34).

In our institution, we have therefore taken the decision to perform RT-PCR SARS-CoV-2 testing by nasopharyngeal swab in a specifically dedicated area to our patients at day −2 or −1 before administration of chemotherapy or immunotherapy treatment, if feasible. The reason is to discover the disease in its pre-symptomatic phase to prevent initiation of a potentially immunosuppressive treatment to diminish chances of major complications and to be able to intensively follow up these patients. In addition, identifying those pre-symptomatic and asymptomatic patients will help rapidly isolate these COVID-19+ patients to prevent further spreading of the disease to health personnel and to other patients. For those patients with respiratory symptoms a chest CT scan is also performed to increase sensitivity to the diagnoses of the disease. In addition, CT pulmonary angiogram is performed for those cases with acute inflammatory syndrome to rule out pulmonary embolism and to initiate intensive anticoagulation therapy. For patients confirmed COVID-19+, systemic treatments are delayed and patient is surveyed or treated accordingly. All COVID-19+ cases will be re-tested at around 15 days from the initial testing. For those patients asymptomatic throughout the infection with a negative test at 15 days, systemic treatment can be initiated. In case of a symptomatic course of the infection, systemic anticancer treatment can be started around 15 days after the end of symptoms. If the second PCR test still is positive, patients need to be rested at 7–15 days and systemic treatment must be delayed if possible. For those cases still COVID-19+ after at least 15 days from the diagnostic PCR, chemotherapy can be initiated if the patient has no symptoms for at least 7 days for those cases where benefit/risk is in favor for cancer therapy. Soon, as validated RT-PCR SARS-CoV-2 testing becomes available in local laboratories, testing will be performed at the same time as the standard pre-chemotherapy blood samples before every cycle of treatment, with results being directly reported to our institution.

In the near future, the availability of laboratory IgM or IgG validated testing to evaluate the previous SARS-CoV-2 exposure will be helpful in order to fully picture the real incidence of this disease in overall population and more particularly in cancer patients. This will become crucial if further studies observe the acquisition of an adaptive immunity capable of preventing re-infection to SARS-CoV-2 infection, or at least to severe forms to allow treating cancer patients without additional risk from COVID-19.

## Author Contributions

AV and MA: oncologist point of view and writing and bibliographic search. SA and LD: radiologist point of view and writing and figure creation. All authors contributed to the article and approved the submitted version.

## Conflict of Interest

The authors declare that the research was conducted in the absence of any commercial or financial relationships that could be construed as a potential conflict of interest.
